# Alcohol use as a predictor of the course of major depressive disorder: a prospective population-based study

**DOI:** 10.1017/S2045796023000070

**Published:** 2023-02-25

**Authors:** Maria J. E. Schouten, Margreet ten Have, Marlous Tuithof, Ron de Graaf, Jack J. M. Dekker, Anna E. Goudriaan, Matthijs Blankers

**Affiliations:** 1 Department of Research, Arkin Institute for Mental Health Care, Amsterdam, the Netherlands; 2 Trimbos Institute – Netherlands Institute of Mental Health and Addiction, Utrecht, the Netherlands; 3 Amsterdam UMC, Department of Psychiatry, University of Amsterdam, and Amsterdam Institute for Addiction Research, Amsterdam Public Health research institute, Amsterdam, the Netherlands; 4 Department of Clinical, Neuro- and Developmental Psychology, Vrije Universiteit Amsterdam, Amsterdam, the Netherlands, Amsterdam Public Health research institute, Amsterdam, the Netherlands

**Keywords:** alcohol abuse, depression, epidemiology, mental health, prospective study

## Abstract

**Aims:**

There are indications that problematic alcohol use may negatively impact the course of major depressive disorder (MDD). However, most studies on alcohol use and adverse MDD outcomes are conducted amongst MDD populations with (severe) alcohol use disorder in psychiatric treatment settings. Therefore, it remains unclear whether these results can be generalised to the general population. In light of this, we examined the longitudinal relationship between alcohol use and MDD persistence after a 3-year follow-up amongst people with MDD from the general population.

**Methods:**

Data were derived from the Netherlands Mental Health Survey and Incidence Study-2 (NEMESIS-2), a psychiatric epidemiological prospective study comprising four waves amongst the adult Dutch general population (*n* = 6.646). The study sample (*n* = 642) consisted of those with 12-month MDD who participated at the follow-up wave. The outcome was 12-month MDD persistence after the 3-year follow-up, which was assessed via the Composite International Diagnostic Interview version 3.0. Weekly alcohol consumption was operationalised as non-drinking (0 drinks), low-risk drinking (⩽7 drinks; reference), at-risk drinking (women 8–13 drinks, men 8–20 drinks) and high-risk drinking (women ⩾14, men ⩾21 drinks). We performed univariate and multiple logistic regression analyses, which were adjusted for various socio-demographic and health-related factors.

**Results:**

The majority (67.4%) of the MDD sample were female, while the mean age was 47.1 years. Amongst these, 23.8% were non-drinkers, 52.0% were low-risk drinkers and 14.3% and 9.4% were at-risk and high-risk drinkers, respectively. Around one-quarter of the sample (23.6%) met the criteria for a persistent MDD after 3-year follow-up. No statistically significant association was found between alcohol use and MDD persistence, either for the crude model or the adjusted models. In comparison to low-risk drinking, the full adjusted model showed no statistically significant associations between MDD persistence and non-drinking (odds ratio (OR) = 1.15, *p* = 0.620), at-risk drinking (OR = 1.25, *p* = 0.423), or high-risk drinking (OR = 0.74, *p* = 0.501).

**Conclusions:**

Contrary to our expectations, our findings showed that alcohol use was not a predictor of MDD persistence after 3-year follow-up amongst people with MDD from the general population.

## Introduction

Depression and alcohol use are two major contributors to morbidity and mortality worldwide (GBD 2016 Mental Disorders Collaborators, 2018; GBD 2019 Alcohol Collaborators, 2022). Many epidemiological studies have found consistent associations between problematic alcohol use and depression (Rehm *et al*., 2017). Problematic alcohol use, including alcohol use disorder (AUD) and other non-clinical levels of hazardous drinking, frequently co-occur with major depressive disorder (MDD) (Hunt *et al*., 2020; Shmulewitz *et al*., 2021). A recent literature review reports the lifetime prevalence of AUD amongst populations with lifetime MDD as ranging from approximately 27% to 40%, whereas the prevalence of MDD in people with AUD in the prior 12-month period ranges from 4% to 22% (Castillo-Carniglia *et al*., 2019). Co-occurring problematic alcohol use and depression has been associated with various negative health outcomes, including, amongst other things, an increased risk of greater AUD severity, suicide attempts, higher disease burden and lower life satisfaction and worse general and social functioning (Sullivan *et al*., 2005; Gadermann *et al*., 2012; Briere *et al*., 2014). Therefore, it is of great clinical and scientific importance to gain greater insight into the associations between risky drinking patterns and MDD.

There are indications that problematic alcohol use may negatively impact upon the course of MDD. Sullivan *et al*. (2005) conducted a systematic review of the prevalence and impact of alcohol problems amongst depressed populations. The influence of alcohol problems on MDD course was examined in only six studies. Two studies found problematic alcohol use to be associated with an increased risk of relapse and a decreased likelihood of recovery from depression. However, three other studies found no association between problematic alcohol use and either relapse or recurrent episodes of depression. The evidence concerning the impact of problematic alcohol use upon depression course is thus inconclusive (Sullivan *et al*., 2005). Closer examination of these studies on alcohol use and MDD course also shows that all six of these aforementioned studies were conducted in psychiatric treatment settings and included people with comorbid MDD and either alcohol abuse or dependence (Sullivan *et al*., 2005). This is problematic insofar as it restricts the range of alcohol use problems, which means that at-risk drinkers may not be included. Moreover, the naturalistic course of MDD is best studied amongst subjects from the general population, as MDD treatment samples might be more prone to selection bias due to overrepresentation of severe MDD cases (Eaton *et al*., 2008). This underscores the need for more prospective population-based studies that examine the relationship between the full range of alcohol use, including non-risk, low-risk, at-risk and high-risk drinking, in addition to unfavourable MDD course.

There is a relative dearth of prospective general population-based studies conducted amongst MDD samples with different levels of alcohol use, such as at-risk and high-risk drinkers. According to Dutch alcohol drinking guidelines, at-risk drinkers are defined as people who drink between 8–13 (women) and 8–20 (men) standard drinks per week, whereas high-risk drinkers consume ⩾14 (women) and ⩾21 (men) drinks on a weekly basis (State of Health and Care, 2022). Most general population-based studies are focused on populations with MDD and AUD, which means there is limited generalisability to other non-AUD MDD populations (de Graaf *et al*., 2002; Alonso *et al*., 2004; Lai *et al*., 2015; Hasin *et al*., 2018; Hunt *et al*., 2020). While several population-based studies have been conducted amongst non-drinking, light, moderate and heavy drinkers, these are invariably restricted to student or young adult drinking populations and focus either on cross-sectional relationships between alcohol use and depressive symptoms or the longitudinal relationships between alcohol use and risk of subsequent depression (Caldwell *et al*., 2002; O'Donnell *et al*., 2006; Gémes *et al*., 2019*a*). In this respect, it therefore remains unclear whether any of the associations that have been found between AUD and MDD course are generalisable to non-clinical drinking patterns in general population-based MDD samples.

The current literature is inconsistent with regard to whether the relationship between alcohol consumption and depression is linear or non-linear in non-clinical alcohol drinking populations. In non-linear relationships, such as U-shaped or J-shaped relations, both non-drinkers (e.g. former drinkers and lifetime abstainers) and high-risk drinkers have an increased risk of depression or depressive symptoms (Rodgers *et al*., 2000; Graham *et al*., 2007; Li *et al*., 2020). These inconsistent findings may be explained as deriving from differences in methodological study design and statistical analysis. Indeed, there are indications that both the way depression and alcohol use outcomes are measured and whether or not the findings are adjusted for confounding factors play a role in whether associations between alcohol intake and depression are found or not (Graham *et al*., 2007; Li *et al*., 2020). This testifies to the importance of both including the full range of drinkers (as well as non-drinkers) in studies and controlling for various potential confounding factors in statistical analyses.

Gaining a better understanding of the longitudinal relationship between different levels of alcohol use and the course of MDD would contribute to the current knowledge base on alcohol use amongst MDD populations. Consequently, we sought to examine the relationship between non-drinking, at-risk drinking and high-risk drinking in comparison to low-risk drinking and MDD persistence after a 3-year follow-up amongst adults with MDD from the general population.

## Methods

### Sample

The data were derived from the Netherlands Mental Health Survey and Incidence Study-2 (NEMESIS-2), a psychiatric epidemiological cohort study amongst individuals aged 18–64 years from the Dutch general population. The first measurement wave (*T*_0_) took place between November 2007 and July 2009. Three follow-up waves (i.e. *T*_1_, *T*_2_ and *T*_3_) took place 3 years after each previous wave (de Graaf *et al*., 2010). The response rate of the first wave was 65.1% (*n* = 6.646). All the respondents from *T*_0_ were approached to take part in the follow-up measurement; the response rate for the subsequent waves, excluding those who were deceased, was 80.4% (*T*_1_, *n* = 5.303), 87.8% (*T*_2_, *n* = 4.618) and 87.7% (*T*_3_, *n* = 4.007), respectively (ten Have *et al*., 2021).

Amongst other instruments, the Composite International Diagnostic Interview 3.0 (CIDI 3.0) was included in the NEMESIS-2 measurement package (Haro *et al*., 2006). The CIDI is a widely used structured lay-administered diagnostic interview, which has demonstrated good validity for common mental disorders like MDD and was developed by the World Health Organization (Kessler and Ustün, 2004; Haro *et al*., 2006). We selected subjects who met the diagnostic criteria for an MDD in the prior 12-month period either at *T*_0_, *T*_1_ or *T*_2_ based on the CIDI 3.0, and participated in the first follow-up wave. Following ten Have *et al*. (2018), subjects with schizophrenia (*n* = 15) were excluded so that the findings could not be attributable to this condition. This resulted in an MDD study sample comprising 642 subjects, from either one of the following time pairs during which the course of MDD was examined: *T*_0_ − *T*_1_, *T*_1_ − *T*_2_ or *T*_2_ − *T*_3_. The response rate within our MDD sample for the time pairs was 79.9% (*n* = 279, *T*_0_ − *T*_1_), 82.7% (*n* = 187, *T*_1_ − *T*_2_) and 80.9% (*n* = 165, *T*_2_ − *T*_3_), respectively. Attrition (also by death) was not significantly associated with any of the drinking groups in the first time pair (non-drinking OR 1.51, *p* = 0.213; at-risk OR 1.12, *p* = 0.771; high-risk OR 0.61, *p* = 0.382). This was also the case in the second time pair (non-drinking OR 0.87, *p* = 0.732; at-risk OR 0.78, *p* = 0.681; high-risk OR 1.06, *p* = 0.925). In the third time pair, a small trend was found among non-drinkers, but these were not statistically significant (non-drinking OR 2.05, *p* = 0.070; at-risk OR 0.80, *p* = 0.708; high-risk OR 0.80, *p* = 0.780).

### Procedures

The respondents were selected through a multistage, stratified random sampling procedure of households. Within each household, the person with the most recent birthday, who was aged between 18 and 64 years old and spoke sufficient Dutch was selected to be interviewed. The majority of the face-to-face interviews were laptop computer-assisted and conducted at the respondents’ homes (de Graaf *et al*., 2010). Those who were insufficiently fluent in Dutch or long-term institutionalised were excluded. The mean duration of the interviews was 95, 84, 83 and 101 min for the interviews at *T*_0_, *T*_1_, *T*_2_ and *T*_3_, respectively (ten Have *et al*., 2021). The NEMESIS-2 study was approved by a medical ethics committee [the Medical Ethics Review Committee for Institutions on Mental Health Care (METIGG)], approval number NL18210.097.07. All the respondents were both verbally informed and provided with written information about the study prior to giving their written informed consent to participate at each wave (de Graaf *et al.*, 2010).

### Measures

#### MDD course

MDD was measured across all four waves using the CIDI 3.0 interview (Haro *et al*., 2006). MDD course was operationalised as the occurrence of any changes in the presence of an MDD diagnosis between measurement waves. We considered MDD persistence to be an unfavourable MDD course. MDD persistence was operationalised as subjects who still met the 12-month MDD criteria after the 3-year follow-up, at the next measurement wave.

#### Alcohol use

Alcohol consumption in the prior 12-month period was measured at all four waves using two CIDI questions: (I) ‘In the past 12 months, how often did you usually have at least one drink (answer categories: every day, nearly every day, 3–4 days a week, 1–2 days a week, 1–3 days a month, or less than once a month)?’ and (II) ‘On the days you drank in the past 12 months, around how many drinks did you usually have per day?’. In light of the aforementioned possibility of a J-shaped association between alcohol intake and depression, alcohol use was considered a categorical variable in our study (Rodgers *et al*., 2000). The participants were categorised into drinking groups based on their alcohol consumption in the 12-month period prior to the interview in which they met criteria for an MDD. We calculated the total number of drinks per week by multiplication and subsequently categorised subjects into the following groups: non-drinking (0 drinks weekly), low-risk drinking (⩽7 drinks weekly), at-risk drinking (8–13 drinks weekly for women and 8–20 drinks weekly for men) and high-risk drinking (⩾14 drinks weekly for women and ⩾21 drinks weekly for men). Both non-drinking and low-risk drinking groups adhere to the Dutch drinking guidelines, which advises not to drink any alcoholic beverages and if one does, to drink no more than one drink each drinking day (Meyboom-de Jong, 2018). The at-risk drinking group are often defined as people who drink above the national drinking guidelines (Case *et al*., 2019), but not excessively. The high-risk drinking group consumes alcohol in an excessive manner from the perspective of the Dutch excessive alcohol use norms (State of Health and Care, 2022).

#### Sample characteristics

We examined various socio-demographic and health-related measures in order to describe the differences between the non-risk, low-risk, at-risk and high-risk drinking groups in the MDD study sample.

*Socio-demographic characteristics* included sex (male/female), age (years), education (primary, lower secondary, higher secondary, higher professional/university), urbanicity of the place of residence (rural/city), living situation (with partner/single) and employment situation (paid job/not in paid employment). These characteristics were measured with self-constructed questions in all four waves, with the exception of education, which was only measured at *T*_0_ and *T*_3_, and was imputed for *T*_1_ and *T*_2_ using baseline values.

*Health-related characteristics* were measured in all four waves and included presence of any comorbid somatic disorders (yes/no), mental health care use (defined as ⩾1 contact made with mental health care services for emotional-, alcohol- or drug-related problems in the last 12 months (yes/no) and any psychotropic medication use in the last 12 months (yes/no)). Furthermore, presence of any anxiety disorder (yes/no), any drug abuse/dependence (yes/no), number of depressive episodes, age of onset of first MDD and the severity of any mental disorder (mild/moderate/severe) were measured using the CIDI 3.0.

*Lifestyle-related characteristics* included tobacco use in the last 4 weeks (yes/no) and body mass index score (BMI), which were both measured in all four assessment waves.

*Vulnerability-related characteristics* were the number of negative life events and the presence of child abuse. The presence of ten negative life events (e.g. divorce, death of a relative) in the prior 12-month period was assessed at all four waves using the Brugha life events section, which has been shown to have good sensitivity and specificity (Brugha and Cragg, 1990). Childhood abuse was operationalised in terms of having experienced prior to the age of 16 any emotional neglect, psychological abuse, physical abuse on ⩾2 occasions or sexual abuse on ⩾1 occasion. The questions used to measure childhood abuse were also used in other studies, such as NEMESIS-1 and the Netherlands Study of Depression and Anxiety (NESDA) (Vogel *et al*., 2018). Childhood abuse was measured at *T*_0_ and imputed for each subsequent follow-up measurement.

*General functioning-related characteristics* were measured at all four waves using the 36-item Short-Form Health Survey (SF-36) (Ware and Sherbourne, 1992; Larson, 1997). The SF-36 is a widely used instrument and includes a multi-item scale that assesses eight health concepts. We used the mental functioning and physical functioning subscales, with a higher total subscale score (0–100) indicating better mental or physical functioning (Stewart *et al*., 1988).

#### Data analysis

All analyses were performed with STATA 16, using two-tailed testing procedures with alpha levels set at 0.05. The MDD study sample included 642 subjects, from either one of the following time pairs on which the course of MDD (i.e. MDD persistence) was examined: *T*_0_ − *T*_1_, *T*_1_ − *T*_2_ or *T*_2_ − *T*_3_. These time pairs were collapsed into one dataset, due to the small number of subjects in the high-risk drinking group in each individual time pair. Data were analysed in a long format using the cluster option (i.e. vce(cluster [subject ID])) to correct for multiple observations within subjects since some participants participated in multiple waves, as was done in Guloksuz *et al*. (2018). Considering that our research question was conducted on MDD sub-samples and aimed to identify associations rather than incidence or prevalence, sampling weights were not applied (Honings *et al*., 2016). All analyses were conducted on the observed data.

For descriptive purposes, we examined the differences in socio-demographic and health-related characteristics between the non-risk, low-risk, at-risk and high-risk drinking groups. First, for every characteristic, we performed a chi-square test (*χ*^2^) for categorical variables or omnibus (*F*) tests for continuous variables. We then selected, based on the statistical significance of these tests (*p* < 0.05), the characteristics to be examined further in the post-hoc analyses using pairwise comparisons and a Bonferroni correction, in order to assess in which specific groups the difference occurred. Furthermore, the selected significant characteristics (based on *χ*^2^ and omnibus testing) were also included as covariates in the main analysis, in order to control for any potential confounding effects.

For the main analysis in which we examined the relationship between alcohol use and MDD persistence after a 3-year follow-up, we carried out both univariate and multiple logistic regression analyses. The univariate regression included the crude model (model 1), whereas the multiple regression models included both the model adjusted for age and gender (model 2) and the full model that was adjusted for all the previously selected characteristics (model 3). We chose the low-risk drinking group as our reference group to align with other alcohol-related studies and because non-drinkers often experience more adverse health outcomes and, as such, are less suitable as a reference group (Rodgers *et al*., 2007; Gémes *et al*., 2019*b*). An additional analysis was performed, using a broader definition for MDD persistence defined as meeting the criteria for MDD in the prior 3-year period (instead of 12-month MDD) at the next wave. The results of this additional analysis were adjusted for all the previously selected characteristics from the post-hoc analyses.

## Results

### Sample characteristics

The study sample comprised 642 subjects with a 12-month MDD. The majority (67.4%) of the MDD sample was female, while the mean age was 47.1 years. Amongst these, 23.8% were non-drinkers, while 52.0%, 14.3% and 9.4% were low-risk, at-risk and high-risk drinkers, respectively. Almost one-quarter of the sample (23.6%) met the criteria for a persistent MDD after the 3-year follow-up interval. Post-hoc analyses showed that statistically significant differences between the drinking groups were found for gender, age, education, unemployment, age of first MDD onset, presence of somatic comorbidities, medication use, mental health care use, BMI, smoking and physical and mental functioning. At-risk and high-risk drinkers were more likely to be male compared to their low-risk and non-drinking counterparts, while low-risk drinkers were typically younger than all the other drinking groups. High-risk drinkers were more frequently engaged with mental health care services than low-risk and at-risk drinkers. Non-drinkers were found to use more medication than low-risk drinkers and, moreover, often had a higher BMI and somatic comorbidities in comparison to all the other drinking groups. This indicates that the non-drinker group experienced worse somatic health than the other drinking groups. See Table 1 for a detailed overview of the descriptive characteristics and post-hoc analyses for each drinking group.

**Table 1.** Descriptive characteristics of non-drinking, low-risk, at-risk and high-risk drinking groups amongst subjects with MDD

	Non-drinking (*n* = 153)		Low-risk drinking (*n* = 334)		At-risk drinking (*n* = 92)		High-risk drinking (*n* = 60)				
	%	(*N*)	%	(*N*)	%	(*N*)	%	(*N*)	*χ*^2^ or *F*	*p*	Post-hoc analyses^a^
*Sociodemographic characteristics*											
Female gender	77.8	(119)	73.1	(244)	47.8	(44)	40.0	(24)	48.9	<0.001	At-risk, high < non-drinking, low
Age [mean, s.d.]	[48.3]	[11.4]	[43.1]	[11.7]	[47.3]	[12.2]	[49.5]	[10.2]	11.0	<0.001	Low < non-drinking; at-risk, high > low
Education									38.7	<0.001	
Primary	11.8	(18)	4.2	(14)	3.3	(3)	0.0	(0)			Low < non-drinking
Lower secondary	37.3	(57)	23.4	(78)	23.9	(22)	36.7	(22)			Low < non-drinking
Higher secondary	30.1	(46)	39.2	(131)	32.6	(30)	41.7	(25)			
Higher professional, university	20.9	(32)	33.2	(111)	40.2	(37)	21.7	(13)			
Single	49.0	(75)	44.9	(150)	46.7	(43)	60.0	(36)	4.8	0.186	
Not in paid employment	50.3	(77)	31.1	(104)	20.7	(19)	41.7	(25)	27.4	<0.001	Low, at-risk < non-drinking; high > at-risk
Living in the city	71.9	(110)	71.9	(240)	67.4	(62)	78.3	(47)	2.2	0.542	
*Health characteristics*											
Any anxiety disorder	39.2	(60)	30.2	(101)	32.6	(30)	21.7	(13)	7.1	0.069	
Depressive episodes [mean, s.d.]	[1.7]	[2.6]	[1.6]	[2.1]	[1.3]	[0.7]	[1.5]	[1.9]	0.8	0.503	
Age first MDD onset [mean, s.d.]	[35.7]	[15.5]	[30.6]	[14.2]	[33.6]	[16.5]	[34.1]	[16.4]	4.5	0.004	Low < non-drinking
Any drug abuse/dependence	7.2	(11)	3.9	(13)	2.2	(2)	3.3	(2)	4.3	0.231	
Any somatic comorbidities	63.2	(96)	45.2	(150)	44.0	(40)	50.0	(30)	14.9	0.002	Low, at-risk < non-drinking
Severity of any mental disorder									11.2	0.082	
Mild	6.5	(10)	6.3	(21)	9.8	(9)	8.3	(5)			
Moderate	28.1	(43)	37.1	(124)	41.3	(38)	23.3	(14)			
Severe	65.4	(100)	56.6	(189)	48.9	(45)	68.3	(41)			
Psychotropic medication use	44.7	(68)	31.9	(106)	38.5	(35)	41.7	(25)	8.2	0.042	Low < non-drinking
Mental health care use	46.1	(70)	39.8	(132)	36.3	(33)	58.3	(35)	9.4	0.024	High > low, at-risk
BMI score [mean, s.d.]	[27.1]	[6.2]	[25.2]	[4.6]	[24.9]	[4.4]	[24.4]	[3.7]	7.3	<0.001	Low, at-risk, high < non-drinking
Smoking	31.6	(48)	39.5	(131)	45.1	(41)	60.0	(36)	15.4	0.001	High > non-drinking, low
*Vulnerability characteristics*											
Any childhood abuse	51.7	(77)	49.5	(163)	47.3	(43)	53.3	(32)	0.74	0.865	
Negative life events [mean, s.d.]	[1.4]	[1.3]	[1.2]	[1.1]	[1.2]	[1.3]	[1.6]	[1.4]	3.1	0.027	n.s.
*General functioning characteristics*											
Physical functioning [mean, s.d.]	[59.7]	[27.2]	[73.4]	[21.2]	[74.2]	[21.3]	[67.6]	[24.3]	13.8	<0.001	Low, at-risk > non-drinking
Mental functioning [mean, s.d.]	[56.2]	[25.4]	[65.9]	[22.4]	[65.3]	[21.3]	[59.3]	[22.3]	7.1	<0.001	Low, at-risk > non-drinking

Total sample includes 642 subjects, three subjects did not report alcohol use; s.d., standard deviation

a Post-hoc analyses with Bonferroni correction, n.s. = no significant differences between groups. Continuous measures: age: in years, depressive episodes: number of depressive episodes, age of fist MDD onset: in years, negative life events: number of negative life events (e.g. divorce, death of a relative) in the previous 12 months, general functioning: mental functioning and physical functioning are subscales of the SF-36 (Short Form (36) Health Survey), a higher total subscale score (0–100) indicates better mental or physical functioning.

### Alcohol use and MDD persistence

We found no statistically significant associations between MDD persistence and non-drinking, at-risk and high-risk drinking in comparison to low-risk drinking, neither in the crude model nor both of the adjusted models (Table 2). Hence, the low-risk drinking group did not differ from the other drinking groups in terms of MDD persistence after the 3-year follow-up. Moreover, in the additional analyses that used a broader definition of MDD persistence (i.e. meeting the criteria for an MDD in the prior 3-year period at the first subsequent measurement wave), no statistically significant associations were found for non-drinking (OR = 0.98, *p* = 0.949), at-risk drinking (OR = 1.19, *p* = 0.492) or high-risk drinking (OR = 1.49, *p* = 0.248) in comparison to low-risk drinking. All in all, alcohol consumption did not appear to be a predictor of MDD persistence amongst people with MDD from the general population.

**Table 2.** Alcohol use and 12-month MDD persistence after a 3-year follow-up

	OR	S.E.	*p*-value	95% CI
	Model 1: crude			
Non-drinking	1.34	0.33	0.248	0.82–2.18
At-risk drinking	1.19	0.32	0.529	0.70–2.01
High-risk drinking	0.85	0.34	0.676	0.39–1.85
	Model 2: adjusted for age and gender			
Non-drinking	1.35	0.34	0.241	0.82–2.21
At-risk drinking	1.16	0.32	0.592	0.67–2.00
High-risk drinking	0.82	0.34	0.640	0.36–1.86
	Model 3: adjusted for various characteristics^a^			
Non-drinking	1.15	0.32	0.620	0.67–1.97
At-risk drinking	1.25	0.34	0.423	0.73–2.15
High-risk drinking	0.74	0.33	0.501	0.31–1.76

Reference group, low-risk drinking group; OR, odds ratio; s.e., standard error; 95% CI, 95% confidence interval

a Based on *χ*^2^ or omnibus *F*-test: gender, age, education, unemployment, age of onset of first MDD, presence of somatic comorbidities, medication use, mental health care use, BMI, smoking, negative life events, physical and mental functioning.

## Discussion

### Key findings

The present study is one of the few prospective population-based studies that examines whether the level of alcohol use is a predictor of MDD persistence after a 3-year follow-up amongst people with 12-month MDD. Our results showed that there were no statistically significant associations between non-drinking, at-risk and high-risk drinking and MDD persistence after the 3-year follow-up in comparison with low-risk drinking. Hence, contrary to our expectations, our study showed that alcohol use was not associated with MDD persistence amongst people with MDD.

### Relevance and implications of the findings

Only a few studies have hitherto examined the relationship between alcohol use and the course of MDD. Our findings are in line with another prospective cohort study, the Netherlands Study of Depression in Older persons (NESDO), amongst elderly people with late-life depression. The NESDO findings showed that, in comparison to non-drinking, there were no statistically significant associations between moderate and at-risk drinking and intermittent depression, as well as for chronic depression after a 2-year follow-up (Bruin *et al*., 2018). Both these findings thus indicate that alcohol use was not a predictor of adverse MDD course amongst MDD populations. Despite this finding, there remains a strong evidence base that underscores the importance of alcohol reduction among problem drinkers, insofar as reduction is associated with various major mental and physical health-related benefits (see Charlet and Heinz (2017) for a detailed overview). Therefore, reducing alcohol use remains critically important from both a public health and clinical perspective, especially for vulnerable groups such as at-risk and high-risk drinkers with a co-occurring MDD.

The majority of population-based studies that have examined alcohol use and the course of MDD have included only AUD populations in their analyses, whilst our study also included non-clinical drinkers. Our study's findings can be said in broad alignment with some of these studies amongst AUD/MDD populations. Two studies focused on AUD amongst people with either a major depressive episode or a mixed sample including people with MDD and/or an anxiety disorder (Boschloo *et al*., 2012; Hoertel *et al*., 2017). The findings of these studies are mixed, insofar as one prospective study found that only severe AUD (i.e. DSM-IV alcohol dependence) and not milder AUD (DSM-IV alcohol abuse) was associated with MDD persistence and/or anxiety disorder persistence after a 2-year follow-up (Boschloo *et al*., 2012). Another large cohort study amongst adult American population did not find AUD to be a predictor of either persistence or the recurrence of a major depressive episode after a 3-year follow-up (Hoertel *et al*., 2017). Finally, two NEMESIS studies also did not find conclusive evidence concerning whether the presence of any comorbid substance use disorder influenced the course of depression. The presence of a remitted substance use disorder at baseline was found to predict the recurrence of MDD, whereas the presence of a current substance use disorder was found to predict the chronicity of MDD after a 6-year follow-up amongst people with MDD (ten Have *et al*., 2018). However, the presence of a substance use disorder was not found to be related to depressive episode duration in either a minor depressive disorder or MDD study samples (ten Have *et al*., 2017). These studies’ findings shed light on both the complexity and high degree of variability of the course of co-occurring AUD and depression (McHugh and Weiss, 2019).

When viewed in conjunction with one another, these inconclusive findings from the extant literature, the relative dearth of general population-based studies examining the impact of different drinking patterns on the course of MDD, and the fact that the aforementioned studies included different sub-populations, underscore the need for further research into this particular topic. Specifically, future studies could explore whether changes in alcohol use (as opposed to the prior year's alcohol use) amongst people with MDD are associated with worse or better depression outcomes. Gaining insight into this matter might strengthen preventive alcohol reduction initiatives. Ideally, future studies could also include excessively drinking MDD populations, as the current evidence points mostly towards a relation between more severe alcohol problems and potential adverse outcomes on MDD course, and because high-risk drinking generally increases the risk of AUD (Grant *et al*., 2017; Patrick *et al*., 2021).

### Strengths and limitations

To the best of our knowledge, no prior work has hitherto examined the relationship between the full range of alcohol consumption and MDD persistence amongst adults with MDD from the general population. Additional strengths of the present study pertain to its use of a prospective design, clinically validated diagnostic interviews and a relatively large study sample.

However, our findings should also be interpreted in light of some limitations. First, we used data from all the NEMESIS-2 waves in order to come to a sufficiently large study sample. Including cases from only the first wave would have led to underpowered analyses, as the number of high-risk drinkers with MDD at baseline was too low. Despite the relatively large study sample, the number of cases in the high-risk drinking group remains modest, insofar as the majority of people with MDD are either non-drinkers or low-risk drinkers. This illustrates both the complexity and challenges associated with examining associations between different drinking groups in general population-based study samples. Moreover, our group of high-risk drinkers may in fact have been a heterogeneous group, including, amongst others, people who irrespective of their frequent alcohol use experienced few problems while others might have had more severe alcohol use problems. While we were unable to conduct subgroup analysed due to the small number of high-risk drinkers in our sample, we cannot rule out the possibility that there might be a subgroup of high-risk drinkers that may experience worse depression outcomes. Second, alcohol consumption was measured using retrospective self-reports, which is the most common method of measuring alcohol consumption in research. However, recall bias may have led to both biased estimates and underreporting of the actual level of alcohol consumption in the prior 12-month period (Stockwell *et al*., 2004; Ekholm *et al*., 2008). Third, assessments were conducted through face-to-face interviews, which may have led subjects to give socially desirable answers regarding their alcohol intake (Bowling, 2005; McKenna *et al*., 2018). Our estimates of alcohol consumption might therefore be on the conservative side. Consequently, the examined relationship between alcohol use and MDD persistence might thus have become attenuated. Fourth, given that people with either insufficient mastery of the Dutch language, without a permanent home address or those who were institutionalised were excluded from the sample procedure (de Graaf *et al*., 2010), findings can not be generalised to these aforementioned groups. Finally, MDD classification was based on the CIDI 3.0, a fully structured diagnostic interview administered by a trained lay interviewer (de Graaf *et al*., 2010). The CIDI has shown good psychometric properties and is considered appropriate for classifying MDD, hence its common use in scientific research (Andrews and Peters, 1998; Kessler and Ustün, 2004; Levis *et al*., 2018). However, a recent individual participant data meta-analysis showed that compared to semi-structured interviews, fully structured interviews (such as the CIDI) tend to classify fewer people with high-level symptoms as having an MDD (Levis *et al*., 2018). The use of the CIDI may thus have influenced our findings, insofar as severe MDD cases may have remained undetected in our sample.

## Conclusion

Compared to low-risk drinking, we found no significant associations between non-drinking, at-risk and high-risk drinking and MDD persistence after a 3-year follow-up amongst people from the general population with MDD. Alcohol use was therefore not found to be a predictor of MDD persistence in our study.

## Data

The data on which this manuscript is based are not publicly available. However, data from NEMESIS-2 are available upon request. The Dutch Ministry of Health financed the data, on the proviso that these data can be used freely under certain restrictions and always under the supervision of the principal investigator (PI) of the study. Thus, some access restrictions do apply to the data. The PI of the study is the second author of this study (MtH) and can at all times be contacted to request the data. At any time, prospective researchers can contact the PI of NEMESIS-2 and submit a research plan, describing the background to the study, research questions, variables to be used in the analyses and an outline of the analyses to be conducted.
